# Supplementary material for: Molecular screening for the mutation associated with canine degenerative myelopathy (SOD1:c.118G > A) in German Shepherd dogs in Brazil
Source: PLoS One. 2020 Nov 16;15(11):e0242347. doi: 10.1371/journal.pone.0242347 (PMC7668602; doi:10.1371/journal.pone.0242347)

S1 Figure. Original version of the gel image shown in Figure 1. Lanes indicated with a red "X" were spliced from the presented figure to evidence the different genotype patterns. The first (top) lane corresponds to a 100bp ladder (Ludwig Biotecnologia LTDA). The fourth (top) lane corresponds to the Swiss Shepherd dog previously confirmed as AA homozygous used as a reference in this study. Fifth and sixty (top) lanes correspond to GG homozygous individuals, and seventh and ninth (top) lanes correspond to AG heterozygous individuals.

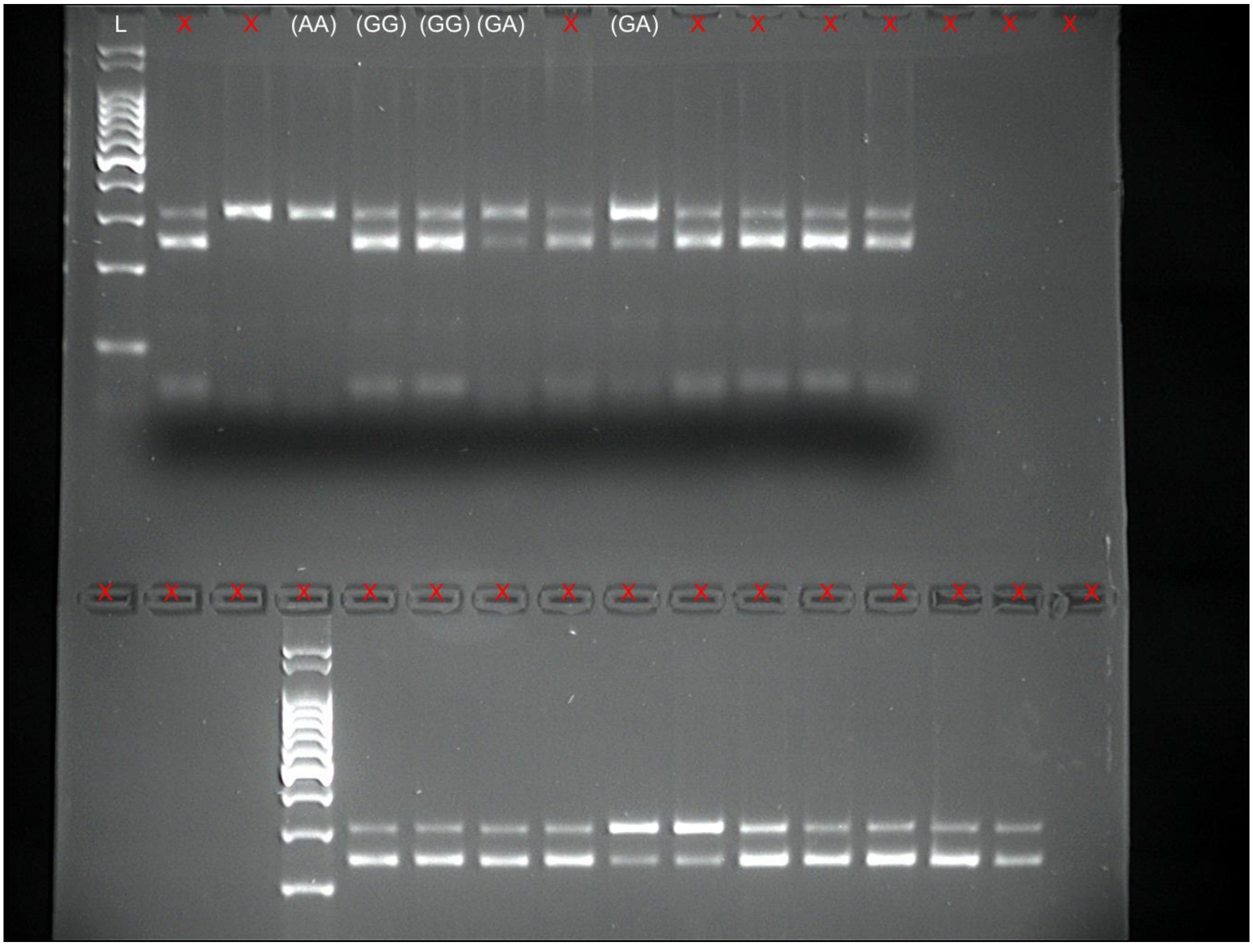

Supplement: S1 Fig — Lanes indicated with a red “X” were spliced from the presented figure to evidence the different genotype patterns. The first (top) lane corresponds to a 100bp ladder (Ludwig Biotecnologia LTDA). The fourth (top) lane corresponds to the Swiss Shepherd dog previously confirmed as AA homozygous used as a reference in this study. Fifth and sixty (top) lanes correspond to GG homozygous individuals, and seventh and ninth (top) lanes correspond to AG heterozygous individuals. (PDF) [file pone.0242347.s001.pdf]
