# Supplementary material for: Molecular screening for the mutation associated with canine degenerative myelopathy (SOD1:c.118G > A) in German Shepherd dogs in Brazil
Source: PLoS One. 2020 Nov 16;15(11):e0242347. doi: 10.1371/journal.pone.0242347 (PMC7668602; doi:10.1371/journal.pone.0242347)

S2 Figure. Partial sequence of the *SOD1* gene indicating the location of the ENSCAFG00000008859:g.26540247del (highlighted in red). Two previous described mutations are highlighted in blue. Exon 2 region is highlighted with yellow background

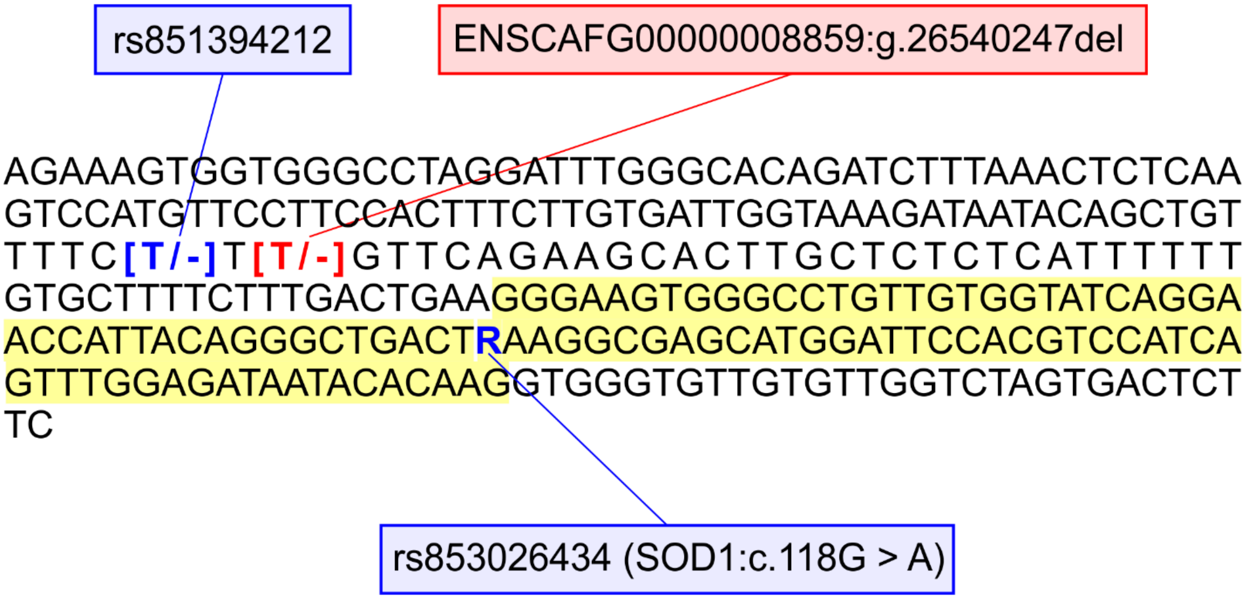

Supplement: S2 Fig — Two previous described mutations are highlighted in blue. Exon 2 region is highlighted with yellow background. (PDF) [file pone.0242347.s002.pdf]
