# Supplementary material for: Molecular screening for the mutation associated with canine degenerative myelopathy (SOD1:c.118G > A) in German Shepherd dogs in Brazil
Source: PLoS One. 2020 Nov 16;15(11):e0242347. doi: 10.1371/journal.pone.0242347 (PMC7668602; doi:10.1371/journal.pone.0242347)
Supplement: S1 Table — (DOCX) [file pone.0242347.s003.docx]

**S1 Table. Signalment, location and genotype of individual dogs enrolled in the present study.**

| **Laboratory record** | **Breeding stock** | **Gender** | **Age (years)** | **State** | **Genotype** |
| --- | --- | --- | --- | --- | --- |
| CFPA0001 | 1 | M | 11 | Santa Catarina | *GG* |
| CFPA0002 | 1 | F | 11 | Santa Catarina | *GG* |
| CFPA0003 | 1 | M | 5 | Santa Catarina | *GG* |
| CFPA0004 | 1 | F | 4 | Santa Catarina | *GG* |
| CFPA0005 | 1 | M | 2 | Santa Catarina | *GG* |
| CFPA0006 | 1 | M | 4 | Santa Catarina | *AG* |
| CFPA0007 | 1 | F | 5 | Santa Catarina | *AG* |
| CFPA0008 | 1 | F | 3 | Santa Catarina | *GG* |
| CFPA0009 | 1 | F | 5 | Santa Catarina | *GG* |
| CFPA0010 | 1 | M | 4 | Santa Catarina | *GG* |
| CFPA0011 | 1 | M | 6 | Santa Catarina | *AG* |
| CFPA0014 | 1 | F | 6 | Santa Catarina | *GG* |
| CFPA0015 | 1 | M | 7 | Santa Catarina | *GG* |
| CFPA0016 | 1 | M | 10 | Santa Catarina | *GG* |
| CFPA0017 | 1 | M | 2 | Santa Catarina | *GG* |
| CFPA0018 | 1 | F | 5 | Santa Catarina | *GG* |
| CFPA0019 | 1 | F | 4 | Santa Catarina | *GG* |
| CFPA0020 | 1 | F | 4 | Santa Catarina | *AG* |
| CFPA0022 | 1 | F | 8 | Santa Catarina | *GG* |
| CFPA0023 | 1 | F | 9 | Santa Catarina | *AG* |
| CFPA0024 | 1 | M | 9 | Santa Catarina | *GG* |
| CFPA0025 | 1 | M | 6 | Santa Catarina | *GG* |
| CFPA0026 | 1 | F | 2 | Santa Catarina | *GG* |
| CFPA0027 | 1 | F | 7 | Santa Catarina | *GG* |
| CFPA0028 | 1 | M | 2 | Santa Catarina | *AG* |
| CFPA0029 | 1 | F | 11 | Santa Catarina | *GG* |
| CFPA0030 | 1 | M | 2 | Santa Catarina | *GG* |
| CFPA0031 | 1 | F | 10 | Santa Catarina | *AG* |
| CFPA0032 | 1 | F | 7 | Santa Catarina | *GG* |
| CFPA0033 | 1 | M | 7 | Santa Catarina | *GG* |
| CFPA0034 | 1 | M | 7 | Santa Catarina | *GG* |
| CFPA0035 | 1 | M | 2 | Santa Catarina | *GG* |
| CFPA0036 | 1 | M | 7 | Santa Catarina | *GG* |
| CFPA0037 | 1 | F | 5 | Santa Catarina | *GG* |
| CFPA0038 | 1 | F | 6 | Santa Catarina | *GG* |
| CFPA0039 | 1 | M | 3 | Santa Catarina | *GG* |
| CFPA0040 | 1 | M | 5 | Santa Catarina | *GG* |
| CFPA0041 | 1 | F | 7 | Santa Catarina | *GG* |
| CFPA0042 | 1 | F | 2 | Santa Catarina | *GG* |
| CFPA0043 | 1 | F | 5 | Santa Catarina | *AG* |
| CFPA0044 | 1 | F | 8 | Santa Catarina | *AG* |
| CFPA0045 | 1 | F | 6 | Santa Catarina | *AG* |
| CFPA0047 | 1 | M | 5 | Santa Catarina | *AG* |
| CFPA0048 | 1 | M | 3 | Santa Catarina | *GG* |
| CFPA0049 | 1 | F | 3 | Santa Catarina | *GG* |
| CFPA0050 | 1 | M | 2 | Santa Catarina | *GG* |
| CFPA0051 | 1 | F | 2 | Santa Catarina | *AG* |
| CFPA0052 | 1 | F | 4 | Santa Catarina | *GG* |
| CFPA0053 | 1 | F | 7 | Santa Catarina | *GG* |
| CFPA0054 | 1 | F | 11 | Santa Catarina | *GG* |
| CFPA0055 | 1 | M | 2 | Santa Catarina | *GG* |
| CFPA0056 | 1 | F | 8 | Santa Catarina | *GG* |
| CFPA0057 | 1 | M | 2 | Santa Catarina | *GG* |
| CFPA0013 | 1 | F | 8 | Santa Catarina | *GG* |
| CFPA0012 | 1 | M | 4 | Santa Catarina | NO |
| CFPA0046 | 1 | F | 1.5 | Santa Catarina | NO |
| CFPA0058 | 2 | M | 5 | Bahia | *GG* |
| CFPA0059 | 2 | M | 5 | Bahia | *AG* |
| CFPA0060 | 2 | M | 2.5 | Bahia | *AG* |
| CFPA0061 | 2 | F | 5 | Bahia | *GG* |
| CFPA0062 | 2 | M | 2.5 | Bahia | *AG* |
| CFPA0063 | 2 | M | 5 | Bahia | *GG* |
| CFPA0064 | 2 | M | 5 | Bahia | *GG* |
| CFPA0065 | 2 | F | 2.5 | Bahia | *AG* |
| CFPA0066 | 2 | F | 4 | Bahia | *GG* |
| CFPA0067 | 2 | M | 5 | Bahia | *GG* |
| CFPA0068 | 2 | M | 5 | Bahia | *GG* |
| CFPA0069 | 2 | M | 5 | Bahia | *GG* |
| CFPA0070 | 2 | M | 5 | Bahia | *GG* |
| CFPA0071 | 2 | M | 5 | Bahia | *AG* |
| CFPA0072 | 2 | F | 7 | Bahia | *GG* |
| CFPA0073 | 2 | M | 2.5 | Bahia | *GG* |
| CFPA0076 | 3 | F | 11 | Bahia | *GG* |
| CFPA0077 | 3 | F | 5 | Bahia | *GG* |
| CFPA0079 | 3 | F | 2 | Bahia | *GG* |
| CFPA0080 | 3 | F | 1 year | Bahia | *GG* |
| CFPA0081 | 3 | M | 6 | Bahia | *GG* |
| CFPA0082 | 3 | M | 7 | Bahia | *GG* |
| CFPA0083 | 3 | F | 1 | Bahia | *AG* |
| CFPA0085 | 3 | F | 1.5 | Bahia | *GG* |
| CFPA0086 | 3 | F | 1.5 | Bahia | *AG* |
| CFPA0087 | 3 | F | 7 | Bahia | *GG* |
| CFPA0088 | 3 | M | 1.5 | Bahia | *GG* |
| CFPA0089 | 3 | F | 7 | Bahia | *AG* |
| CFPA0090 | 3 | F | 2.5 | Bahia | *GG* |
| CFPA0091 | 3 | F | 10 | Bahia | *GG* |
| CFPA0094 | 3 | F | 5 | Bahia | *GG* |
| CFPA0096 | 3 | F | 6 1 year | Bahia | *GG* |
| CFPA0078 | 4 | M | 1 year | Bahia | *GG* |
| CFPA0084 | 4 | M | 1 year | Bahia | *AG* |
| CFPA0092 | 4 | M | 1 year | Bahia | *AG* |
| CFPA0093 | 4 | M | 1 year | Bahia | *GG* |
| CFPA0095 | 4 | M | 1 year | Bahia | *AG* |
| CFPA0097 | 4 | M | 1 year | Bahia | *GG* |
| CFPA0021 | 5 | F | 7 | Pernambuco | *GG* |
| CFPA0075 | 5 | M | 8 | Pernambuco | *GG* |
| CFPA0098 | 5 | M | 13 | Pernambuco | *GG* |

NO, undetermined.
